# A new species of *Siamspinops* Dankittipakul & Corronca, 2009 from Guizhou, China (Araneae, Selenopidae)

**DOI:** 10.3897/BDJ.11.e102450

**Published:** 2023-04-18

**Authors:** Cheng Wang, Jiahui Gan, Mi Xiaoqi

**Affiliations:** 1 Guizhou Provincial Key Laboratory for Biodiversity Conservation and Utilization in the Fanjing Mountain Region, Tongren University, Tongren, China Guizhou Provincial Key Laboratory for Biodiversity Conservation and Utilization in the Fanjing Mountain Region, Tongren University Tongren China

**Keywords:** East Asia, morphology, taxonomy, wall crab spider

## Abstract

**Background:**

The wall crab spider genus *Siamspinops* Dankittipakul & Corronca, 2009 contains eight species restricted to South and Southeast Asia, of which two are recorded from China.

**New information:**

A new species, *Siamspinopsyejiei* **sp. n.**, is diagnosed and described based on both sexes from Foding Mountain National Nature Reserve, Guizhou, China. Diagnostic photos of the habitus and copulatory organs and a distributional map are provided.

## Introduction

The selenopid spider genus *Siamspinops* Dankittipakul & Corronca, 2009 is diagnosed by the presence of at least 11 ventral spines on tibiae Ⅰ and Ⅱ and seven ventral spines on metatarsi Ⅰ and Ⅱ (Dankittipakul and Corronca 2009). It is represented by eight species primarily distributed in tropical areas of South and Southeast Asia, including two recorded from China. Although all of the species are known from diagnostic illustrations, six of them are known only from original descriptions and half of them are known only from females, indicating that further taxonomic studies and more collecting efforts over a broader area are necessary (World Spider Catalog 2023).

In our recent study of the samples collected from Foding Mountain, two selenopid species have been found. One was identified as *Selenopsbursarius* Karsch, 1879 and the other was identified as an undescribed species of the genus *Siamspinops*, which is described and illustrated herein. The discovery has revealed that *Siamspinops* is not restricted to tropical areas and that there may be undiscovered diversity in temperate regions.

## Materials and methods

All specimens are deposited in the museum of Tongren University, Tongren, China. The specimens were examined with an Olympus SZX10 stereomicroscope. After dissection, the epigynes were cleared in a trypsin enzyme solution before examination and imaging. The left male palp was used for the description and illustrations. Photographs of the copulatory organs and habitus were taken with a Kuy Nice CCD mounted on an Olympus BX43 compound microscope. Compound focus images were generated using Helicon Focus v. 6.7.1.

Measurements are given for the female holotype and male paratype in millimetres. Leg measurements are given as: total length (femur, patella, tibia, metatarsus, tarsus). References to figures in the cited papers are listed in lowercase type (i.e. fig. or figs.); figures in this paper are noted with an initial capital (i.e. Fig. or Figs.). Palp terminology follows those of Lin et al. (2022) and epigyne terminology follows those of Crews (2023). Abbreviations used in the text and figures are as follows: **AB** accessory bulb; **ALE** anterior lateral eye; **AME** anterior median eye; **C** conductor; **CD** copulatory duct; **CO** copulatory opening; **dRTA** dorso-retrolateral tibial apophysis; **E** embolus; **EP** epigynal pocket; **FD** fertilisation duct; **MA** median apophysis; **ML** median lobe; **PLE** posterior lateral eye; **PME** posterior median eye; **vRTA** vento-retrolateral tibial apophysis; **S** spermatheca.

## Taxon treatments

### Siamspinops yejiei

Wang, Gan & Mi sp. n.

464A7040-6D47-548F-8AC1-429B5FF11270

8E69025B-3A3D-43D3-BFC7-31EA2B5CD7CA

#### Materials

- **Type status:** Holotype. **Occurrence:** sex: female; occurrenceID: 0E1ACA16-D1C7-548F-A801-BEA5051675B1; **Taxon:** scientificName: *Siamspinopsyejiei*; **Location:** country: China; stateProvince: Guizhou; county: Shiqian; locality: Ganxi Township, Fuyan Village; verbatimElevation: 800-900 m; verbatimLatitude: 27°21.46′N; verbatimLongitude: 108.2.03′E; **Identification:** identifiedBy: Cheng Wang; **Event:** year: 2020; month: June; day: night of 20; habitat: stone wall
- **Type status:** Paratype. **Occurrence:** sex: 1 male, 1 female; occurrenceID: 0373C481-7FF2-520B-BBC9-9F083D3ED418; **Taxon:** scientificName: *Siamspinopsyejiei*; **Location:** country: China; stateProvince: Guizhou; county: Shiqian; locality: Ganxi Township, Fuyan Village; verbatimElevation: 800-900 m; verbatimLatitude: 27°21.46′N; verbatimLongitude: 108.2.03′E; **Identification:** identifiedBy: Cheng Wang; **Event:** year: 2020; month: June; day: night of 20; habitat: stone wall

#### Description

**Female** (Holotype, TRU-SN-005, Fig. 2A, C, E, F, H; paratype, TRU-SN-006, Fig. 2B, D). Total length 7.47. Carapace 2.94 long, 3.28 wide. Abdomen 4.79 long, 3.70 wide. Eye sizes: AME 0.16, ALE 0.09, PME 0.17, PLE 0.21. Leg measurements: I 9.70 (2.80, 1.15, 2.65, 2.10, 1.00), II 11.75 (3.75, 1.30, 3.20, 2.50, 1.00), III 12.75 (4.05, 1.30, 3.95, 2.45, 1.00), IV 9.90 (3.50, 1.00, 2.45, 2.00, 0.95). Carapace pear-shaped, yellow to brown, with slightly elevated cephalic region bearing a pair of oblique brown stripes behind PMEs and oval thoracic region bearing pairs of irregular, brown patches mediolaterally. Chelicerae yellow, each with three promarginal and two retromarginal teeth. Endites slightly longer than wide. Labium almost trapeziform. Sternum almost oval, with straight anterior margin, covered with brown setae. Legs pale to yellow, mingled with green-brown patches, with three (1-1-1) dorsal spines, two (1-1-0) prolateral spines on femora Ⅰ and 15 (1-2-2-2-2-2-2-2) and 10 (2-2-2-2-2) ventral spines on tibiae and metatarsi Ⅰ and Ⅱ, respectively. Abdomen elongate-oval, dorsum grey, mingled with dark brown spots and patches, covered with short setae; venter pale to brown, with dark margin. Epigyne (Fig. 2A–D): longer than wide, with broad, postero-marginally located pocket and sub-oval lobe in median field; copulatory openings beneath postero-lateral atrial margins; accessory bulb straight, bar-shaped, anteriorly extending; copulatory ducts long, strongly convoluted, forming six or seven coils; spermathecae elongated, obliquely extending.

**Figure 1.**
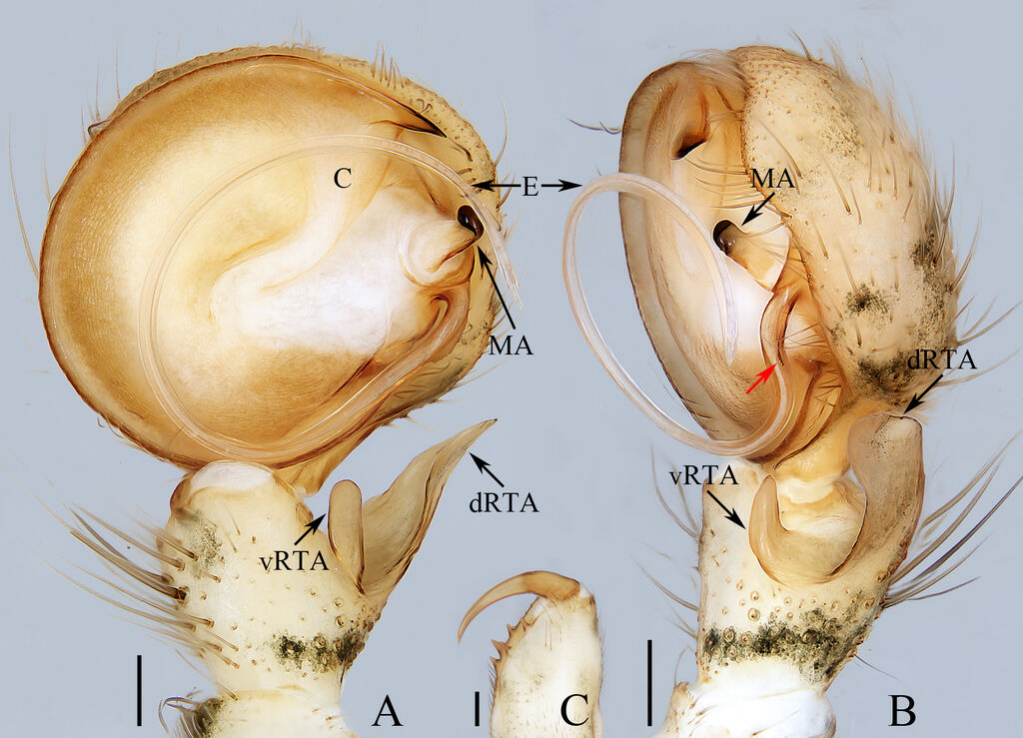
Male palp and chelicera of *Siamspinopsyejiei* sp. n., paratype. **A** palp, ventral; **B** ditto, retrolateral; **C** chelicera, posterior. Scale bars: 0.2 mm.

**Figure 2.**
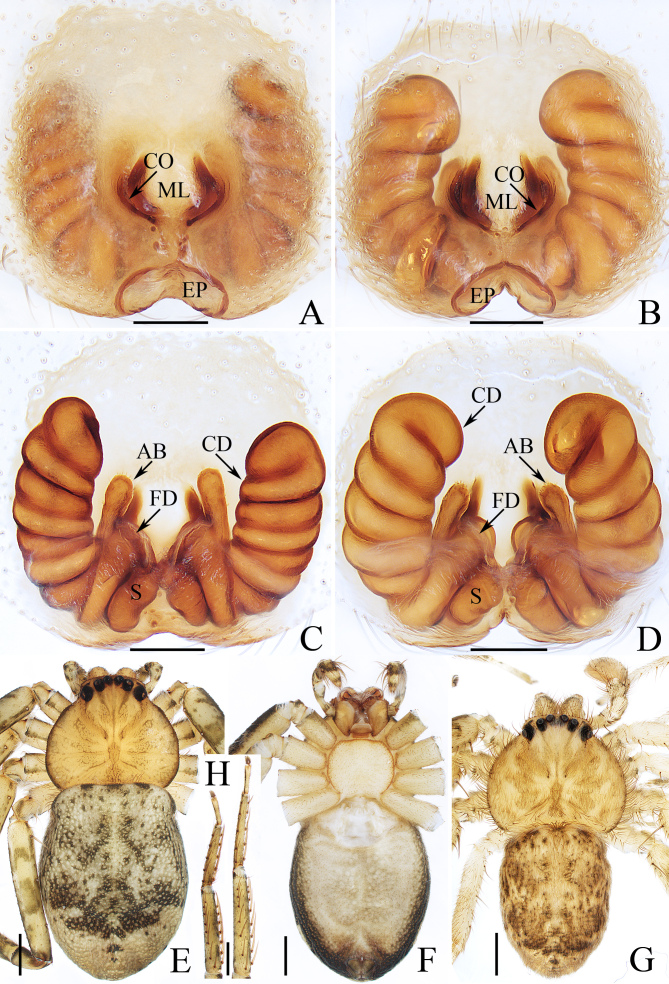
*Siamspinopsyejiei* sp. n., **A, C, E, F, H** female holotype; **B, D** female paratype; **G** male paratype. **A, B** epigyne, ventral; **C, D** vulva, dorsal; **E, G** habitus, dorsal; **F** ditto, ventral; **H** tibiae, metatarsi and tarsi of left legs I and II, ventral. Scale bars: 0.2 mm (**A–D**); 1.0 mm (**E–H**).

**Male** (paratype, TRU-SN-007, Fig. 1A–C, Fig. 2G). Total length 6.30. Carapace 2.9 long, 3.2 wide. Abdomen 3.74 long, 2.7 wide. Eye sizes: AME 0.16, ALE 0.09, PME 0.16, PLE 0.20. Leg measurements: I 9.90 (2.85, 1.15, 2.65, 2.10, 1.15), II 11.90 (3.70, 1.25, 3.25, 2.50, 1.20), III 13.15 (4.00, 1.25, 4.25, 2.40, 1.25), IV 9.60 (2.80, 1.15, 2.50, 2.15, 1.00). Habitus similar to that of female, but paler and covered with dense setae on carapace and only with 13 ventral spines on tibiae Ⅰ and 14 on tibiae II. Palp (Fig. 1A, B): tibia slightly longer than wide, bearing long setae retrolatero-dorsally, with straight, apically blunt vRTA about equal in length to the width of dRTA in ventral view and flat, tapered dRTA with pointed tip directed towards about 45 degrees in ventral view; cymbium setose; bulb flat, almost round; conductor almost T-shaped, tip sclerotised, pointed; median apophysis anteroprolateral to embolic base, curved inwards distally; embolus curved into a circle, with small, serrated processes at base.

#### Diagnosis

*Siamspinopsyejiei* sp. n. closely resembles *S.banna* Lin & Li, 2022 in having a very similar epigynal pocket and male palp, but it can be distinguished by the following: 1) the accessory bulb (described as spermathecal head in Lin et al. (2022)) does not extend anteriorly beyond the copulatory ducts in dorsal view (Fig. 2C, D), versus extends beyond the copulatory ducts in *S.banna* (Lin et al. 2022: fig. 36B); 2) the epigynal pocket is about 1/3 the epigynal width (Fig. 2A, B), versus about 1/2 the epigynal width in *S.banna* (Lin et al. 2022: fig. 36A); 3) the embolus has serrated processes at the base (Fig. 1B, see the red arrow), versus absent in *S.banna* (Lin et al. 2022: fig. 35B); 4) the median apophysis is widest at the base in ventral view (Fig. 1A), versus widest medially in *S.banna* (Lin et al. 2022: fig. 35A); 5) tip of the conductor is prolateral to the retrolateral margin of bulb in ventral view (Fig. 1A), whereas it is retrolateral to the retrolateral margin of bulb (Lin et al. 2022: fig. 35A); 6) the male chelicera has two retromarginal teeth (Fig. 1C), versus just one in *S.banna* (see the description in Lin et al. (2022)).

#### Etymology

The species is named after Mr. Yejie Lin, who guided us through the method of taking photographs and helped with species identification; noun (name) in genitive case.

#### Distribution

**Figure 3.**
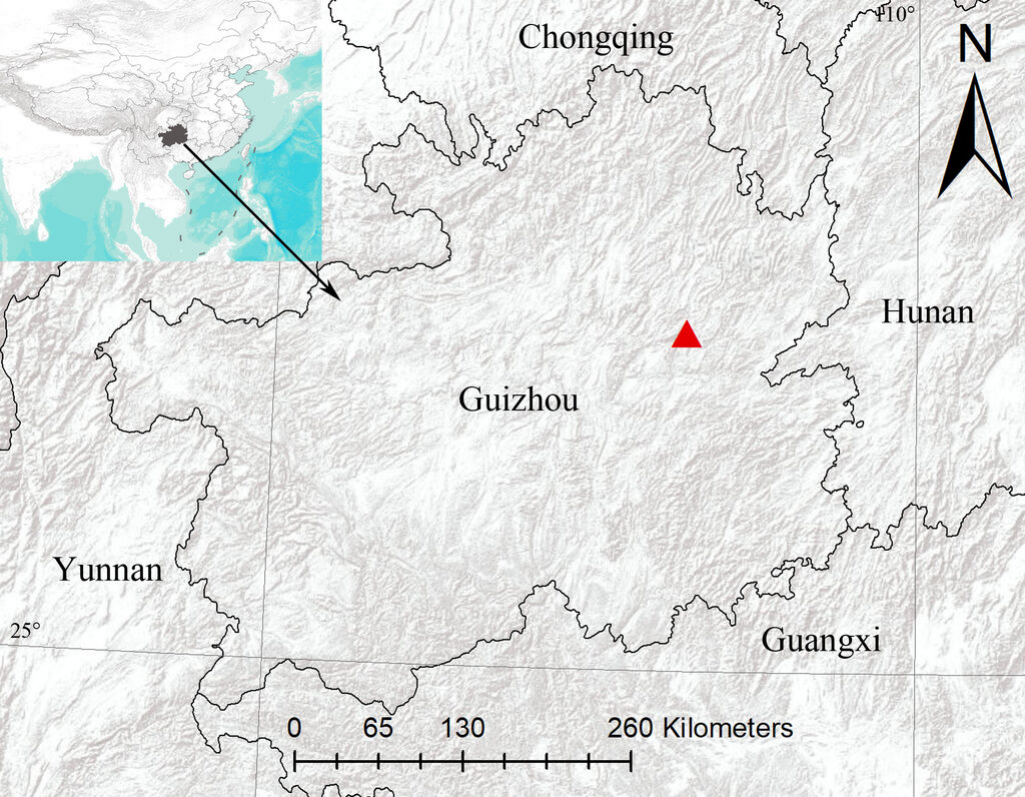
The type locality of *Siamspinopsyejiei* sp. n.

China (Guizhou) (Fig. 3).

## Supplementary Material

XML Treatment for Siamspinops yejiei
